# **The role of high sensitive C-reactive protein and histopathological evaluation in chronic gastritis patients with or without Helicobacter pylori infection** ^**1**^

**DOI:** 10.1590/s0102-865020190030000010

**Published:** 2019-03-21

**Authors:** Eren Altun, Ayla Yildiz, Celalettin Cevik, Gulay Turan

**Affiliations:** I Assistant Professor, Balikesir University, Faculty of Medicine, Department of Pathology, Balikesir, Turkey. Scientific, intellectual, conception and design of the study; acquisition of data; technical procedures; histopathological examinations; manuscript preparation and writing; critical revision.; II Assistant Professor, Balikesir University, Faculty of Medicine, Department of Gastroenterology, Balikesir, Turkey. Conception and design of the study.; III Assistant Professor, Balikesir University, Faculty of Health Sciences, Balikesir, Turkey. Acquisition, analysis and interpretation of data; histopathological examinations; statistics analysis.

**Keywords:** Gastritis, C-Reactive Protein, Helicobacter pylori, Inflammation

## Abstract

**Purpose:**

To evaluate serum levels of high-sensitivity C-reactive protein (hs-CRP) in chronic gastritis patients to predict Helicobacter pylori (HP) infection, inflammatory activity, and precancerous lesions.

**Methods:**

A total of 811 patients with upper gastrointestinal symptoms and histopathological diagnosis of chronic gastritis were enrolled in the study. On endoscopy, five gastric biopsies were taken according to Modified Sydney protocol, which were stained with hematoxylin & eosin and Giemsa

**Results:**

HP infection was found in 28.6% of patients, being significantly more common in specimens with acute and chronic inflammatory activity. Mucosal atrophy, intestinal metaplasia, and dysplasia were found in 20.2%, 18.8% and 2.7% of biopsy specimens. Mean hs-CRP was 1.9±1.6 mg/dl for males and 2.2±1.9 mg/dl for females. hs-CRP average were significantly higher in patients with severe acute inflammation (p:0.049), in patients with severe chronic inflammation (p:0.015) and in those with HP (p: 0.001) . The severity of HP infection increased significantly with the increased degree of acute inflammation, chronic inflammation and hs-CRP level (p=0.001 for both).

**Conclusion:**

Serum hs-CRP level increases in patients with chronic gastritis, it could be an indicator of severity of acute or chronic mucosal inflammation, and presence of HP infection. Therefore, hs-CRP may aid the diagnosis of chronic gastritis, but it is not associated with pre-cancerous lesions.

## Introduction

 Helicobacter pylori (HP) affects over half of the population worldwide and is the major etiology of chronic gastritis^1^. It colonizes in gastric mucosa and causes chronic mucosal inflammation leading to gastritis. HP-associated chronic gastritis is considered precancerous and has a high potential of progression into gastric cancer unless effectively treated^2^. The histolopathological evidence of precancerous lesions of chronic gastritis, such as gastric atrophy and intestinal metaplasia has been well known to be associated with HP infection^3^ ^,^ ^4^.

 Since it is an infection causing an inflammatory disease, HP gastritis is associated with the increase in serum markers of inflammation such as interleukin family, C-reactive protein (CRP), platelets, neutrophils^5^ ^-^ ^8^. Among these markers, CRP is an acute-phase protein, which is commonly used as a marker of various systemic acute and chronic inflammatory conditions^9^. As serum level of CRP is normally less than 1 mg/dl, it can increase up to 35-40 mg/dl in response to infection or inflammation. Upon understanding its value in diagnosis of various conditions, high-sensitivity CRP (hs-CRP) assay has been developed to measure CRP in a sensitivity of 0.5 mg/dl^10^. In addition to being an indicator of inflammation, hs-CRP has been suggested to be a marker of cardiovascular diseases and gastric cancer in patients with HP-associated chronic gastritis^6^ ^,^ ^11^. Although serum level of hs-CRP has been known to increase in chronic gastritis with HP infection, limited studies have focused on the association of hs-CRP level with inflammatory activity and presence of precancerous lesions in biopsy specimens^11^ ^,^ ^12^. If hs-CRP level is predictive of inflammation level and precancerous lesions, it can be used as a simple and easily accesible laboratory test in early diagnosis, treatment response monitoring, and cancer screening in chronic gastritis patients.

 Therefore, in this study we aimed to determine serum levels of hs-CRP in chronic gastric patients with prediction of HP infection and precancerous lesions in a large series of 811 patients. We also investigated the association of severity of HP infection with hs-CRP level and chronic inflammation activity. 

## Methods

###  Ethics approval and consent to participate 

 In this study, the investigation protocol was in accordance with the Helsinki Committee requirement and was approved by the institutional ethical committee of Balikesir University (Decision no: 2018/101, 2018/05/09). Additional patient consent for this clinical research was not required.

###  Study population 

 A total of 811 patients who applied to our clinic with upper gastrointestinal symptoms suggestive of chronic gastritis and underwent endoscopic examination between 2014 and 2017 were enrolled in this study. Patients having active infection, tumor, other gastrointestinal diseases, severe systemic diseases, using any drug with potential effect on gastrointestinal or coagulation system, and pregnant or breastfeeding women were excluded from study.

###  Endoscopy, histopathology and biochemistry 

**Figure 1:**
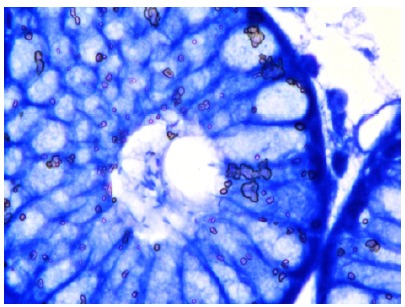
Helicobacter pylori (HP) bacilli seen at the center of gland (Giemsa, x100).

 The data regarding for this retrospective research study has been obtained from Balikesir University Hospital. For endoscopic examination, standard gastrointestinal video endoscopy was performed under sedation and local anesthesia of pharyngeal mucosa. Esophagus, stomach and duodenum were evaluated for the visible abnormal findings and lesions, and four gastric biopsies specimens were taken from antrum and corpus for histopathological diagnosis of gastritis and detection of HP. Biopsy specimens were stained with hematoxylin eosin (H&E) and Giemsa, and evaluated for histopathology and HP under light microscope (Fig. 1). 

 Histopathological findings were defined and classified according to updated Sydney system^3^. Accordingly, gastritis is defined as the atropic or non-atropic inflamation of the gastric mucosa. The presence of mucosal atrophy, which is the loss of specialized glands and decrease in the thickness of mucosa; intestinal metaplasia, which is the metaplastic transformation of gastric glandular and surface epithelium towards intestinal mucosal elements; dysplasia, which is the neoplastic transformation of epithelial cells were evaluated. Acute inflammation or active disease was defined as neutrophilic infiltrates of the lamina propria, pits, or surface epithelium. It was graded as mild if less than one third of pits and surface was infiltrated, moderate if one third to two thirds was infiltrated, and severe if more than two third was infiltrated according the Modified Sydney Protocol. Chronic inflammation was defined as increased lymphocytes and plasma cells in the lamina propria, and graded as mild, moderate or severe according to the density of increase. HP density was defined as mild if organism covered less than one third of the surface, severe if there were larger clusters or a continuous layer over two thirds of surface, moderate if density was somewhere between mild and severe HP density. hs-CRP were measured by standard laboratory methods from intravenous blood sample of each patient.

###  Biochemistry 

 White blood cell count (WBC), platelet count (PC), lymphocyte count (LC), and hs-CRP were measured by standard laboratory methods from intravenous blood sample of each patient. LC/WBC ratio and PC/WBC ratio were calculated as an indicator of HP infection and outcome of patients, respectively.

###  Statistical analysis 

 The study data were summarized by descriptive statistics (e.g., mean, standart deviation, frequency, percentage). Student’s t test or analysis of variance (ANOVA) was used to evaluate the significance of difference in serum hs-CRP level between two or more than two subgroups of study parameters. For comparison of qualitative data between histological severity groups of HP infection, chi-square test was used. Logistic regression analysis was used to evaluate the association of HP with chronic inflammation activity and serum hs-CRP level. A p value smaller than 0.05 was considered statistically significant.

## Results

 On histopathological evaluation, acute and chronic inflammation in any severity was detected in 31.4% and 64% of patients, respectively. Of 811 patients, 28.6% had HP infection. Mucosal atrophy, intestinal metaplasia, and dysplasia were found in 20.2%, 17.8%, and 2,7% of biopsy specimens obtained from 811 patients. 

###  Serum hs-CRP levels 

 Mean hs-CRP was 1.9±1.6 mg/dl for males and 2.2±1.9 mg/dl for females. hs-CRP average; (p: 0.009) were significantly higher in patients with severe acute inflammation (p: 0.049), in patients with severe chronic inflammation (p:0.015) and in those with HP (p: 0.001). Presence of mucosal atrophy, intestinal metaplasia or dysplasia had no significant effect on serum hs-CRP level (p>0.05) (Table 1).

**Table 1:** Demographic and clinical characteristics of study patients (n=811).

		n (%)	hs-CRP (mg/dl) Mean ± SD	p value
Gender	Male	361 (44.5)	1.9±1.6	0.009 ^a^
	Female	450 (55.5)	2.2±1.9	
Years	18-45	538 (66.3)	2.3±1.8	0.006 ^a^
	46 +	273 (33.7)	1.8±1.6	
Acute inflammation	None	556 (68.6)	2.1±1.7	0.049 ^b^
	Mild	153 (18.9)	2.0±1.8	
	Moderate	53 (6.5)	2.3±1.8	
	Severe*	49 (6.0)	2.8±1.9	
Chronic inflammation	None	292 (36.0)	2.2±1.8	0.015 ^b^
	Mild	288 (35.5)	1.9±1.7	
	Moderate	149 (18.4)	2.0±1.6	
	Severe*	82 (10.1)	2.6±2.0	
HP infection	Yes	232 (28.6)	2.5±1.9	*0.001* ^a^
	No	579 (71.4)	2.0±1.7	
Mucosal atrophy	Yes	164 (20.2)	2.1±1.7	0.511 ^a^
	No	647 (79.8)	2.1±1.8	
Intestinal metaplasia	Yes	144 (17.8)	2.0±1.7	*0.417* ^a^
	No	667 (82.2)	2.1±1.8	
Dysplasia	Yes	22 (2.7)	2.0±1.7	0.591 ^a^
	No	789 (97.3)	2.1±1.8	

^a^ Student’s t test, ^b^ ANOVA

hs-CRP, high sensitive C-reactive protein; SD, standard deviation.

###  HP infection 

 When histological severity of HP subgroups were compared for study parameters, we found that severity of HP infection increased significantly with the increased degree of acute inflammation, chronic inflammation and hs-CRP level (p=0.001 for both). We found that severity of HP infection decreased significantly with the increased patient’s age. (p:0.030) (Table 2).

**Table 2:** Severity of HP infection with respect to study parameters (n=811).

		Histological severity of HP infection				
		None (n=708)	Mild (n=109)	Moderate (n=99)	Severe (n=58)	p value
Gender	Male	251 (69.5)	51 (14.1)	38 (10.5)	21 (5.8)	0.159 ^a^
	Female	328 (72.9)	41 (9.1)	50 (11.1)	31 (6.9)	
Years	18-45	183 (67.0)	31 (11.4)	42 (15.4)	17 (6.2)	0.030 ^a^
	46 +	396 (73.6)	61 (11.3)	46 (8.6)	35 (6.5)	
Acute inflammation	None	474 (85.3)	38 (6.8)	24 (4.3)	20 (3.6)	*0.001 ^a^*
	Mild	75 (49.0)	29 (19.0)	34 (22.2)	15 (9.8)	
	Moderate	18 (34.0)	13 (24.5)	15 (28.3)	7 (13.2)	
	Severe	12 (24.5)	12 (24.5)	15 (30.6)	10 (20.4)	
Chronic inflammation	None	283 (96.9)	6 (2.1)	3 (1.0)	0 (0.0)	*0.001 ^a^*
	Mild	190 (66.0)	35 (12.2)	37 (12.8)	26 (9.0)	
	Moderate	81 (54.4)	31 (20.8)	20 (13.4)	17 (11.4)	
	Severe	25 (30.5)	20 (24.4)	28 (34.1)	9 (11.0)	
Mucosal atrophy	Yes	113 (68.9)	20 (12.2)	15 (9.1)	16 (9.8)	0.217 ^a^
	No	466 (72.0)	72 (11.1)	73 (11.3)	36 (5.6)	
Intestinal metaplasia	Yes	97 (67.4)	16 (11.1)	23 (16.0)	8 (5.6)	0.184 ^a^
	No	482 (72.3)	76 (11.4)	65 (9.7)	44 (6.6)	
Dysplasia	Yes	17 (77.3)	3 (13.6)	2 (9.1)	0 (0.0)	0.185 ^a^
	No	562 (71.2)	89 (11.3)	86 (10.9)	52 (6.6)	
hs-CRP (mg/dl)	mean±SD	1.2±0.7	2.1±0.6	2.5±0.2	2.8±0.3	0.001 ^b^

^a^ Chi-square test, ^b^ ANOVA

SD: Standart Deviation

 On the other hand, severity subgroups of HP or the presence of HP infection did not show significance difference with gender, mucosal atrophy, intestinal metaplasia, and dysplasia (p>0.05) (Table 2). 

###  Association of HP infection with chronic inflammation and hs-CRP level 

 According to the multiple regression analysis using the Backward model, gender, age, and severity of HP significantly affect hs-CRP levels, whereas acute inflammation, chronic inflammation, and intestinal metaplasia did not affect hs-CRP levels (Table 3). 

**Table 3:** Evaluation of the relationship between hs-CRP (mg/dl) levels and demographic and clinical findings.

			Standardized Coefficients	t	p	95.0% Confidence Interval for B	
	Beta	Std. Error	Beta			Lower	Upper
(Constant)	2.305	0.116		19.835	0.000	2.077	2.534
Gender	-0.317	0.126	-0.087	-2.525	**0.012**	-0.564	-0.071
Years	-0.405	0.133	-0.106	-3.046	**0.002**	-0.666	-0.144
Acute inflammation	0.106	0.102	0.051	1.042	0.298	-0.094	0.307
Chronic inflammation	-0.064	0.091	-0.034	-0.698	0.485	-0.243	0.115
Severity of HP	0.280	0.076	0.143	3.669	**0.001**	0.130	0.429
Intestinal metaplasia	-0.186	0.167	-0.039	-1.115	0.265	-0.514	0.142

F:5.781 (p:0.001).

 HP status and effective factors were evaluated by logistic regression model. It is observed that the model (c2=676.957, p=0.001, Nagelkerke R2=0.41) is to significant. Sex, acute inflammation, chronic inflammation, and hs-CRP were statistically significant in the model. The correct classification rate of the model in general is 84.2%. According to these results, it is seen that the established model is a valid and usable model. 

 The Presence of HP in those aged between the age of 18 and 45 is 1.72 times (1.15-2.55) higher than those aged 46 years and upper. 

 The presence of HP is higher in patients with low acute inflammation was 2.63 times (1.68-4.11) in patients with moderate acute inflammation 4.2 times (2.0-8.8), and in patients with severe acute inflammation 6.1 times (2.5-14.9) when compared with those without acute inflammation.

 The presence of HP is higher in patients with low levels of chronic inflammation 13.0 times (6.25-27.33), in those with moderate chronic inflammation 13.1 times (5.84-29.52), in patients with severe chronic inflammation 17 times (6.40-45.60) when compared with those without chronic inflammation. The possibility of the presence of HP increases 1.19 times, when 1 unit increases in hs-CRP level.

## Discussion

 In this extensive retrospective research study, we examined endoscopic biopsy specimens and serum hs-CRP levels in 811 patients. We found that in patients acute inflammation, chronic inflammation, and HP infection, hs-CRP levels were above normal levels. As acute and chronic inflammation increases, the level of hs-CRP increases. However, we have indicated that precancerous lesions, metaplasia and atrophy are not associated with the level of hs-CRP. 

 We determined in our study population with chronic gastritis that the mean of hs-CRP was 1.9±1.6 mg/dl for males and 2.2±1.9 mg/dl for females, which was higher 1 mg/dl than the normal hs-CRP level^9^. Consistent with to the studies in literature, it is found increased hs-CRP levels in parallel to the severity of HP gastritis compared to gastritis without HP infection in our study^6^ ^,^ ^12^. In adition, we also found that serum hs-CRP level of chronic gastritis patients with HP infection was significantly higher than those without HP infection and there was significant association between severity of HP infection and hs-CRP level. We also found that serum hs-CRP levels have been affected by the severity of acute or chronic inflammation. Furthermore, Raut *et al.* ^6^ evaluated 80 patients and found that patients with chronic gastritis and HP infection was higher hs-CRP levels than chronic gastritis patients without HP. Similarly in a study of 239 patients with chronic gastritis, Rahmani *et al.* ^12^ reported that serum hs-CRP levels of patients with HP gastritis was higher than those without HP and hs-CRP was positively correlated with mucosal inflammatory activity and dysplasia.

 Although several staining methods have been suggested to determine the severity of HP infection^13^ ^,^ ^14^, H&E, alone or in combination with other methods, is the standard staining technique to detect the presence and extent of infection^3^ ^,^ ^15^. Thus, in the present study, we used the standard H&E staining and Giemsa staining for histopathological evaluation and HP assessment of biopsy materials. In our study population, chronic gastritis was mostly associated with HP infection. It has been known that the presence of acute or chronic inflammatory gastritis is highly predictive of active HP infection in correlation with the severity of inflammation^14^. Similarly, in our study, severity of HP infection increased with the increased severity of acute or chronic inflammation, and in ordinal logistic regression analysis, HP was associated with degree of chronic inflammation activity.

 In clinical studies and meta-analysis, eradication of HP decreases the development of gastric cancer in chronic gastritis patients with precancerous lesions such as atrophy, metaplasia, and displasia^16^ ^,^ ^17^. In addition to causing gastric cancer, HP gastritis has been claimed to induce endothelial dysfunction and potentially to cause cardiovascular pathologies^6^. For early diagnosis against HP and precancerous lesions, histopathological diagnosis of gastritis along with gastric cancer screening is of great importance. In order to standardize the histopathologic diagnosis and classification of gastritis, the Sydney System updated in 1994 which has been commonly used in practice and clinical studies^3^. The updated Sydney System highlighted the importance of combining etiology, topography and histology of gastritis for the best clinical management of the disease^3^ ^,^ ^18^ ^,^ ^19^. However, there has been limited studies on the gastric cancer screening in chronic gastritis patients with HP infection. Chung *et al.* ^11^ reported that hs-CRP levels can be used in combination with other serum markers for screening of gastric cancer in patients with chronic gastritis. Rahmani *et al.* ^12^ found that although the presence of atrophy or metaplasia did not affect serum hs-CRP levels, gastritis patients having dysplasia had significantly higher hs-CRP level than patients without dysplasia. In our study, mucosal atrophy, intestinal metaplasia and dysplasia have no effect on serum hs-CRP level. In contrary to previous studies^3^ ^,^ ^4^, we found that there is no relation between the presence of any precancerous lesion and severity of HP infection. 

 Moreover, H. Pylori is one of the most prevalant chronic infection diseases in the World and previous studies have been clearly demonstrated that there was closely association between peptic ulcer, chronic gastritis, gastric cancer and mucosa associated tissue lymphoma (MALT)^20^. On the other hand, it is known that the presence of extragastrointestinal diseases including coronary arter diseases and diabetes mellitus were higher in patient with H. Pylori infection^21^. In light of these knowledge, the presence of severe H. Pylori infection may accompany high hsCRP levels. 

## Conclusions

 In conclusion, in the literature, commonly serum hs-CRP level have been claimed that it can be used as a simple and easily accesible laboratory test to predict inflammatory activity and precancerous lesions. Thus for treatment response monitoring and cancer screening in chronic gastritis patients could be easy. In this extensive study, we studied the endoscopical biopsy specimens and serum hs-CRP levels of 811 patients with chronic gastritis.

 We indicated that serum hs-CRP level increases in patients with chronic gastritis and this could be an indicator of severity of acute or chronic mucosal inflammation, and presence of HP infection. Therefore, hs-CRP level may aid the diagnosis of chronic gastritis, but is not usefull for predicting cancer screening or precancerous lesions.
